# Compact Bidirectional Promoters for Dual-Gene Expression in a Sleeping Beauty Transposon

**DOI:** 10.3390/ijms21239256

**Published:** 2020-12-04

**Authors:** Kevin He, S. M. Ali Hosseini Rad, Aarati Poudel, Alexander Donald McLellan

**Affiliations:** Department of Microbiology and Immunology, University of Otago, Dunedin 9016, New Zealand; heke6247@student.otago.ac.nz (K.H.); aarati.poudel@postgrad.otago.ac.nz (A.P.)

**Keywords:** Sleeping Beauty transposon, bidirectional promoters, gene expression, gene therapy, synthetic biology, RPBSA, EF-1α, LMP2/TAP1

## Abstract

Promoter choice is an essential consideration for transgene expression in gene therapy. The expression of multiple genes requires ribosomal entry or skip sites, or the use of multiple promoters. Promoter systems comprised of two separate, divergent promoters may significantly increase the size of genetic cassettes intended for use in gene therapy. However, an alternative approach is to use a single, compact, bidirectional promoter. We identified strong and stable bidirectional activity of the RPBSA synthetic promoter comprised of a fragment of the human Rpl13a promoter, together with additional intron/exon structures. The Rpl13a-based promoter drove long-term bidirectional activity of fluorescent proteins. Similar results were obtained for the EF1-α and LMP2/TAP1 promoters. However, in a lentiviral vector, the divergent bidirectional systems failed to produce sufficient titres to translate into an expression system for dual chimeric antigen receptor (CAR) expression. Although bidirectional promoters show excellent applicability to drive short RNA in Sleeping Beauty transposon systems, their possible use in the lentiviral applications requiring longer and more complex RNA, such as dual-CAR cassettes, is limited.

## 1. Introduction

Bidirectional promoters allow transcription from both the sense and antisense direction within a region defined as <1 kb apart [1,2]. In the human genome, 10% of intergenic promoters are classed as bidirectional, often driving genes in a divergent fashion [2,3,4]. CpG islands in promoter regions favour bidirectional activity, with CpG-rich regions in human promoters likely evolutionarily selected as intrinsically bidirectional elements [5]. Most transcripts arising from divergent promoter activity are non-overlapping, while a minority may drive the expression of transcripts that form RNA–RNA duplexes with a role in gene silencing [6,7]. Other divergent, non-coding RNAs (pancRNAs) may upregulate transcription of the opposite transcript via demethylation and likely act to enforce tissue-specific gene expression [6,8].

Surprisingly, the use of naturally occurring bidirectional promoters in synthetic biology has largely been overlooked and separate, divergently placed dual promoters are commonly used in available vectors [9]. The enhancer regions of many naturally occurring promoters may be too distantly placed to allow bidirectional promoters to be feasibly incorporated into expression vectors. Other potentially bidirectional promoters may require the addition of a minimal proximal promoter region to drive expression in the reverse direction [10].

Compact bidirectional promoters would be useful for synthetic biology with potential use in gene therapy [11]. Genetic circuits that require the long-term expression of two or more transgenes could be achieved using such bidirectional promoters. We investigated the utility of bidirectional transgene expression for driving stable gene expression within a genome-integrated Sleeping Beauty system. While developing a Tet-On Sleeping Beauty system for another study [12], we noted strong interference from an RPBSA promoter placed divergently and directly upstream of the Tet-On promoter. Removal of the RPBSA promoter negated this retrograde interference (unpublished data). After further bioinformatic analysis, RPBSA was chosen for further study of bidirectional activity due to an enrichment of transcription factors associated with bidirectional activity [13]. As the majority of transcription binding sites are clustered in the RPL13 proximal promoter region of RPBSA [13], we further examined whether deletion of the intron and exon would affect the strength of bidirectional activity. Results were also compared to the EF1α and LMP2/TAP1 promoters, also described to exhibit bidirectional activity [14,15]. The EF1α promoter has recently been shown to produce optimal function of human CAR T cells for cytokine secretion and anti-tumour cytotoxicity [13]. 

Our findings highlight the potential of bidirectional promoters for driving genome-integrated gene expression in the Sleeping Beauty system. However, our findings suggest that the investigated bidirectional promoters are sub-optimal for the lentiviral production of longer RNA encoding longer dual-CAR constructs.

## 2. Results

### 2.1. Selection of Promoters with Predicted Bidirectional Activity

**Figure 1:**
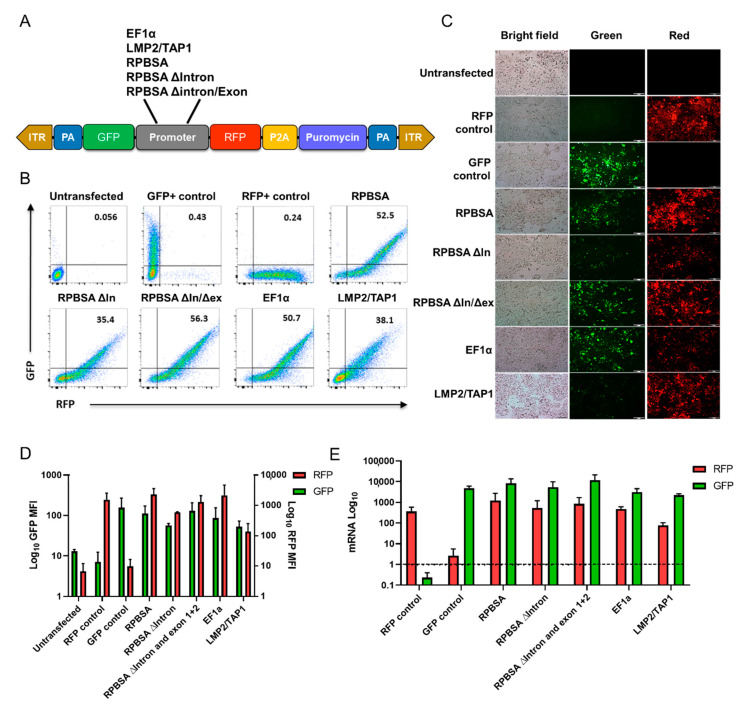
Determination of bidirectional activity promoters in HEK293T. (**A**) Schematic illustration of the Sleeping Beauty backbone bearing five promoters (EF1α, LMP2/TAP1, RPBSA WT, RPBSA ΔIntron, RPBSA ΔIntron/Exon) for driving RFP-P2A-Puromycin expression. T cells. Cells were subjected to FSc and SSc doublet discrimination. (**B**,**C**) Flow cytometric analysis and fluorescence microscopy of HEK293T cells expressing green and red fluorescent proteins (GFP and RFP). (**D**) Assessment of the mean fluorescence intensity (MFI ) of GFP and RFP expression for each promoter. (**E**) Genomic DNA was extracted from cell lysates of Sleeping Beauty transposed HEK293T and qPCR was performed using GFP and RFP primers and β-actin as a housekeeping gene. Data are representative of two to three experiments performed.

Dual fluorescent constructs containing multiple promoters (Figure 1A and Figure S1) were transiently transfected into HEK293T seven days prior to analysis. The RPBSA promoter was confirmed to express transcripts in both the sense and antisense directions. However, upon deletion of the intron, expression levels of both RFP and GFP were reduced. Interestingly, upon further deletion of exons 1 and 2, expression of GFP and RFP was similar to that observed for full-length RPBSA levels or higher. EF1α and LMP2/TAP1 promoters’ bidirectional activity was also confirmed in this setting, as shown previously by Charkravarti et al. and Wright et al., respectively [15,16]. To control for transfection efficiency, we co-transfected an Ametrine-expressing (BFP) vector together with the bidirectional promoter vectors in a 1:1 ratio (Figure S2). The variation in activity of the bidirectional promoters appeared to relate to the transfection efficiency, rather than intrinsic variation in promoter activity within each cell (Figure S2). 

### 2.2. Long-Term Activity of Bidirectional Promoters In Vitro

**Figure 2:**
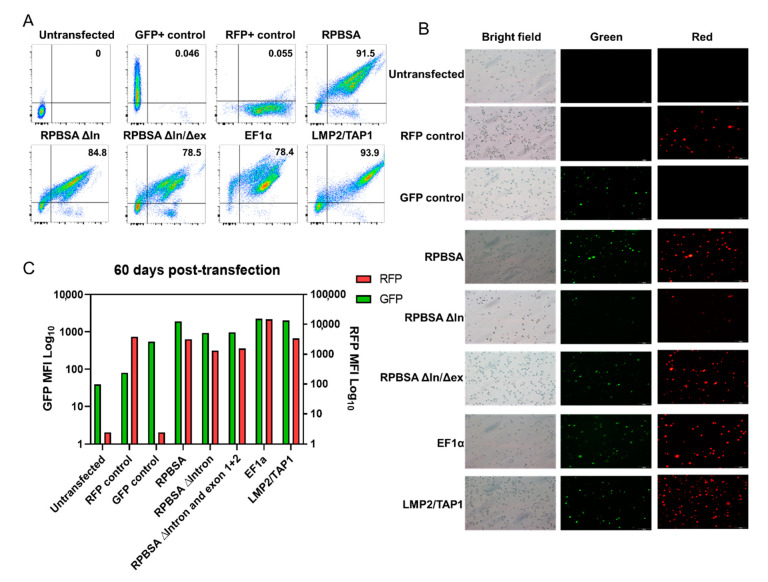
Monitoring maintenance of long-term expression by bidirectional promoters after 60 days. (**A**) Flow cytometry carried out to measure the expression GFP and RFP in Jurkat cells. Cells were subjected to FSc and SSc doublet discrimination. (**B**) Fluorescent microscopy of Jurkat cells expressing GFP and RFP. (**C**) MFI assessment of Jurkat cells for GFP and RFP expression.

To determine if the gene expression by the promoters could be maintained for extended periods, dual fluorescent promoter constructs (Figure 1) were transposed into the Jurkat T cell line using Sleeping Beauty reporter vector [7] and cells maintained for up to 60 days. Stably transposed cells were then analysed by fluorescence microscopy and flow cytometry at days 30 and 60 (Figure S3 and Figure 2). Deletion of the RPBSA ∆Intron 1 resulted in a reduction in bidirectional activity similar to that obtained with short-term culture of HEK293T cells. Interestingly, further deletion of exons 1 and 2 did not restore function in Jurkat cells. EF1α and LMP2/TAP1 promoters maintained robust bidirectional activity. Interestingly, between day 30 and day 60, bidirectional activity in all promoters was reduced, with a proportion of the population showing silenced fluorescent marker expression (Figure 2 and Figure S3). Full-length RPBSA showed the lowest reduction in MFI and was therefore selected for further experiments. Similarly, the LMP2/TAP1 promoter also maintained bidirectional activity, with minimal loss of expression after 60 days, and was also selected for further experiments for driving longer transcripts. 

**Figure 3:**
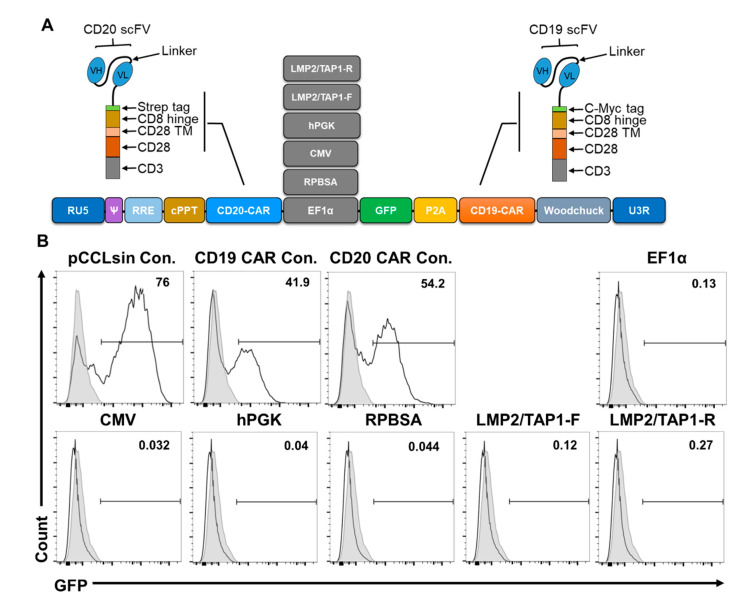
The effect of bidirectional promoters in producing functional lentiviral particles. (**A**) Schematic illustration of the pCCLsin backbone bearing six different internal promoters (LMP2/TAP-1R, LMP2/TAP-1-F, hPGK, CMV, RPBSA, EF1α) for driving GFP-P2A-CD19CAR in the sense direction and CD20CAR in the antisense orientation. (**B**) A representative experiment for the transduction efficiency of primary T cells for the six lentivectors. CD3/CD28 stimulated human primary T cells were transduced at MOI 40 and cells were analysed for GFP expression at 72 h post-transduction by flow cytometry. Results are representative of three independent T cell donors (see text for mean and range of titres obtained for the single and dual constructs).

We next investigated the potential of the bidirectional promoters to drive dual expression of chimeric antigen receptors (CAR) in T cells. CAR T cell therapy can lead to the clonal expansion of malignant cells with lost CD19 expression [17,18]. A solution is to express two CAR targeting both CD19 and CD20 to mitigate immune escape variants that avoid destruction by mono-antigen targeted CAR T cell therapy [17]. We therefore tested the activity of selected promoters RPBSA, EF1α, LMP2/TAP1, as well as hPGK [11] for driving dual CD19 and CD20 CARs in the forward and reverse direction, respectively, using the lentiviral system (Figure 3). Although single CD19 or CD20 CAR controls gave consistently high titres (8.6 × 10^7^–1.34 × 10^8^; mean 1.1 × 10^8^ ± 2.4 × 10^7^ SD; *n* = 2) and 40–80% transduction frequencies, concentrated LV preparations of all dual-CAR constructs yielded low titres (range 1.9 × 10^5^–6.3 × 10^5^; mean 4.1 × 10^5^ ± 1.47 × 10^5^ SD; *n* = 6) and did not result in detectable GFP expression in transduced primary T cells (Figure 3). 

## 3. Discussion

In this study, we compared bidirectional promoters for the optimal expression of two genetic cassettes in HEK293T and Jurkat T cell lines. To determine the strength of the selected bidirectional promoters, we utilized a dual fluorescent vector and measured the bidirectional activity using fluorescent light microscopy, flow cytometry and qPCR using HEK293T and Jurkat cells. In HEK293T, RPBSA was the strongest bidirectional promoter. The deletion of the intron resulted in a reduction in expression, but surprisingly, when both the intron and exon were deleted, expression from the truncated RPBSA promoter became similar to full-length in HEK293T cells. This suggests that, in the case of RPBSA in HEK293T cells, the activation effects of the intron are negated by an inhibitory effect of the exon on the promoter function. In contrast, deletion of the intron or intron plus exon 1 and exon 2 resulted in a drop in bidirectional gene expression in Jurkat cells. 

EF1α was also shown to be a strong bidirectional promoter, especially in the Jurkat T cell line. EF1α is around twice the size of RPBSA and contains an abundance of TF sites together with an enhancer sequence [9,13,14,15]. Although LMP2/TAP1 was determined to be weaker than RPBSA in HEK293T cells (Figure 1), its optimal activity may require IFN-mediated signalling. 

While there is no consensus to determine whether a promoter is bidirectional, structural core elements may exist amongst bidirectional promoters. These include a collection of elements such as the TATA box, CCAAT box, B recognition element (BRE), initiator element (INR) and downstream promoter element (DPE). The TATA box exists in both unidirectional and bidirectional promoters; however, bidirectional promoters tend to have a higher ratio of CCAAT boxes and the BRE element than unidirectional promoters, while the ratio of DPE and INR remains largely unchanged [1]. Although TATA boxes are less frequent in naturally-occurring bidirectional promoters, two of the compact promoters investigated here predicted two TATA boxes each (EF-1 and RPBSA); however, LMP2/TAP1 was predicted to lack TATA boxes [13,19].

Gene therapy almost invariably requires gene expression for extended periods of time, either to maintain therapeutic gene expression to correct inborn errors of cellular function or to clear residual malignant cells [20]. In Jurkat T cells, full-length RPBSA maintained long-term expression of RFP and GFP. EF1α was the strongest promoter, but similar to RPBSA, it also experienced a detectable reduction in expression in both orientations. In contrast, bidirectional activity of the LMP2/TAP1 promoter expression was minimally reduced at day 60. LMP2/TAP1 proteins are involved with the antigen processing and loading onto MHC-I and hence are essential to a fully functioning MHC-I mediated immune response [16,21]. It is therefore possible that the sequence of the LMP2/TAP1 promoter has been selected to be relatively resistant to silencing. The possibility to induce the LMP2/TAP1 with interferon signalling offers a potential way to manage promoter activity in a controllable fashion. 

For anti-cancer CAR T cell therapy, the expression of two gene products separated by an internal ribosome entry site (IRES) or 2A ribosomal skip site is possible, along with the use of dual separate promoters, or double-transduction procedures [13,22]. However, it would be advantageous to express dual-CAR cassettes from both the sense and the antisense direction using a single, compact, bidirectional promoter. Lentiviral vectors show the highest transduction/transfection rate to date and are the gold standard for CAR T cell production [23]. Strategies that reduce the size of the construct while maintaining transduction efficiency are preferred [17]. However, the size and repetitive elements of the lentiviral cassette can impact upon titres and transduction frequencies [24,25,26,27,28]. Multiple transduction protocols may be expensive and labour intensive and produce complexities in the quality control of transduced patient cells [29]. 

While the three promoters showed strong bidirectional activity driving short RNA encoding fluorescent markers in the Sleeping Beauty system, we were not able to produce sufficient titres of lentiviral constructs incorporating bidirectional promoters to allow primary T cell transduction. Therefore, there is little clinical utility in using these bidirectional promoters to drive a dual-CAR T cell phenotype. Our recent comparison of single promoter constructs in driving short or long RNA did reveal major differences in the genomic titres or transduction efficiencies of EF1α, CMV, RPBSA or hPGK. Therefore, the reasons for the poor titres of the dual-CAR constructs could be: (i) the additional genetic elements in the context of the bidirectional promoters interfering with the production of genomic LV RNA [28]; (ii) exosome complex, or cytoplasmic Dicer-mediated, destruction of dsRNA/stem loop structures resulting from shared elements in the two CAR [28] or (iii) alternatively, the repetitive genetic elements of the dual-CAR affecting the reverse transcription of the RNA upon transduction [26,27]. 

Despite the disappointing performance of the dual-CAR constructs in the lentivirus system, the bidirectional constructs developed here offer a compact and convenient method to express two transcripts of at least 700 bp using the Sleeping Beauty transposon system.

## 4. Materials and Methods 

### 4.1. Promoters and Vector Construction

The dual-reporter tdTomato (RFP)/enhanced green fluorescent protein (GFP) plasmid named pSBbiRG in this study was constructed by amplifying the RFP and GFP genes from pSBbiRP and pSBtet-GP [7,9]. The RFP gene was amplified using primers to create 5′ NcoI and 3′ NotI sites, while the GFP gene was amplified with 5′ NheI and 3′ HindIII sites. RFP and GFP were cloned into the backbone of bidirectional pSBbiRP Sleeping Beauty plasmid, removing the existing EF1 promoter. Promoters were amplified with 5′ NcoI sites and 3′ NheI sites from respective plasmids: EF-1 and RPBSA from Sleeping Beauty (pSBbiRP), CMV from pcDNA3.1(-), hPGK from pRRLSIN.cPPT.PGK-GFP.WPRE or LMP2/TAP1 promoter from peripheral blood mononuclear cell cDNA and ligated between RFP and GFP. Total cellular RNA was extracted 48 h after transfection using NucleoSpin RNA Plus Kit (Macherey-Nagel, Düren, Germany) and reverse transcribed using PrimeScript™ RT Reagent Kit (Takara Bio, San Jose, CA, USA). RT PCR was performed using internal primers GFP-Fwd: AGGACGACGGCAACTACAAG, GFP-Rev: TTGTACTCCAGCTTGTGCCC, RFP-FWD: CTTCAAGGTGCGCATGGAG and RFP-REV: TCGAAGTTCATCACGCGCTC for comparison with β-actin Fwd: CTTCCTTCCTGGGCATG and β-actin1 Rev: GTCTTTGCGGATGTCCAC. Promoters were screened for transcription factors associated with bidirectional promoters according to Orekhova et al. [1] using the PROMO ALGGEN database.

### 4.2. Cell Line Transfection and Primary Cell Transduction with Lentiviral Vectors

Cell lines were cultured in a humidified atmosphere at 37 °C with 5% CO_2_. HEK293T (ATCC CRL-1573) and Jurkat E6.1 (ATCC TIB-152) cell lines were cultured in high glucose-DMEM and RPMI-1640, respectively, supplemented with 10% foetal calf serum (FCS; Pan-Biotech GmbH, Aidenbach, Germany), penicillin (100 U/mL) and streptomycin (100 µg/mL) (Gibco). HEK293T cells at 4 × 105 cells/mL in DMEM without antibiotics were added into a 24-well plate and incubated overnight at 37 °C and 5% CO_2_. Lipofectamine 3000 reagent diluted in OptiMEM (Invitrogen, Carlsbadb, CA, USA, #100022058) was added to 100 ng of pCMV(CAT)T7-SB100 (transposase containing plasmid) and 400 ng of Sleeping Beauty plasmid OptiMEM and incubated at room temperature before being added dropwise to HEK293T cells. The cells were then incubated overnight before the medium was replaced with fresh DMEM without antibiotics. Jurkat cells were washed once in PBS, resuspended in Buffer R (Invitrogen #MPK10025) at 2 × 107 cells/mL and electroporated in 100 µL Neon gold tips with 1 µg of pCMV(CAT)T7-SB100, plus 4 µg of the plasmid of interest. The cell:plasmid mix was added to the electroporation tube holder containing 3 mL of electrolyte buffer. Cells were electroporated with three pulses at 1350 V for 10 ms and then added to pre-warmed RPMI-1640 with 10% FCS without antibiotics in a 6-well plate. Lentiviral production and titration on HEK293 with 8 µg/mL of polybrene (Sigma-Aldrich, St. Louis, MI, USA) was carried out using LV-Max Viral production system (ThermoFisher, Waltham, MA, USA, #A35684) as previously described [13]. Peripheral blood mononuclear cells (PBMCs) were obtained from donors with written consent under approval from the University of Otago Human Ethics Committee. Lentiviral transduction of primary T cells was carried out as previously described [13]. Frozen PBMCs were thawed and then rested overnight in T cell expansion media (Thermofisher, Waltham, MA, USA, #A1048501) supplemented with 50 U/mL of hIL-2 (Peprotech, Rocky Hill, NJ, USA, #200-02), L-glutamine and 10 U/mL penicillin and streptomycin (Gibco). CD4 and CD8 T cells were isolated using EasySep Human T cell isolation kit (STEMCELL Technology, Vancouver, BC, Canada, #17951) and T cells activated with Dynabeads Human T-Activator CD3/CD28 (ThermoFisher, # 111.32D) prior to transduction with lentiviral vectors at day 2 [13]. 

### 4.3. Fluorescence Microscopy, Flow Cytometry and qPCR

Brightfield, GFP and RFP images were captured using an Olympus IX-71 inverted microscope. Flow cytometric data were acquired using a BD LSRFortessa with BD FACSDiva software. Data were analysed with FlowJo v10.6.2 software. Events were filtered using FSc/SSc doublet discrimination.

## 5. Conclusions

The bidirectional promoters characterized in this study will be useful for synthetic biology applications, with a potential role in gene therapy using Sleeping Beauty vectors.

## Supplementary Materials

The following are available online at https://www.mdpi.com/1422-0067/21/23/9256/s1, Figure S1: Plasmid construction, Figure S2: Co-transfection of pSB-BFP with bi-directional promoter constructs, Figure S3. Monitoring maintenance of long-term expression by bidirectional promoters after 30 days.

## Abbreviations

EF1α	Eukaryotic translation elongation factor 1 alpha
ITR	Inverted terminal repeats
LMP2	Low molecular mass polypeptide 2
RFP	Red fluorescent protein
RPL13a	Ribosomal protein L13a
RPL41	Ribosomal protein L41
SB	Sleeping Beauty
TAP	Transporter associated with Antigen Processing
