# Supplementary material for: Compact Bidirectional Promoters for Dual-Gene Expression in a Sleeping Beauty Transposon
Source: Int J Mol Sci. 2020 Dec 4;21(23):9256. doi: 10.3390/ijms21239256 (PMC7731152; doi:10.3390/ijms21239256)
Supplement: Supplementary file 1 [file ijms-21-09256-s001.pdf]

Supplementary Figures

He *et al*. Compact bidirectional promoters for dual-gene expression in a Sleeping Beauty transposon

Figure S1

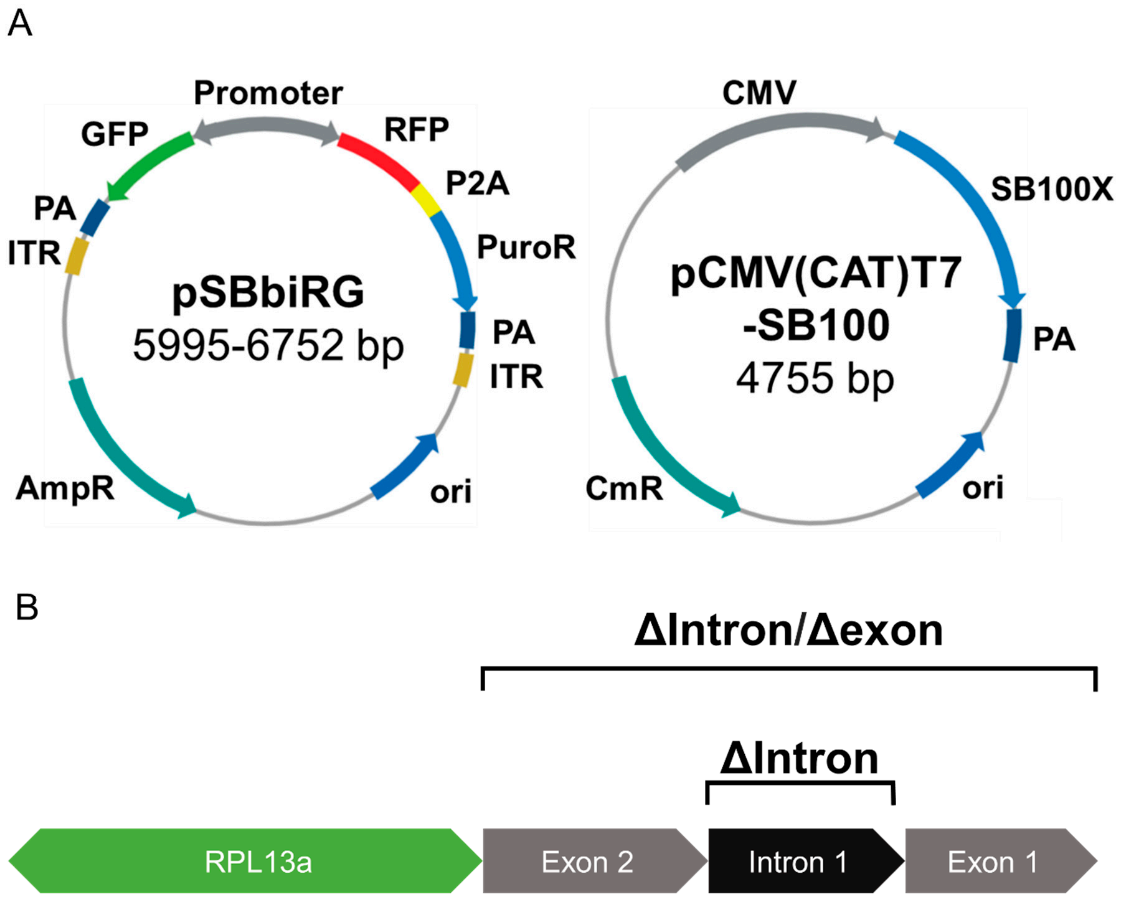

Figure S1. Plasmid construction. A) A plasmid map illustrating the SB plasmid bearing the bidirectional construct or the transposase gene on a separate plasmid, respectively. B) RPBSA as constructed and designed by Kowarz et al. Deletions to RPBSA were made as indicated.

Figure S2

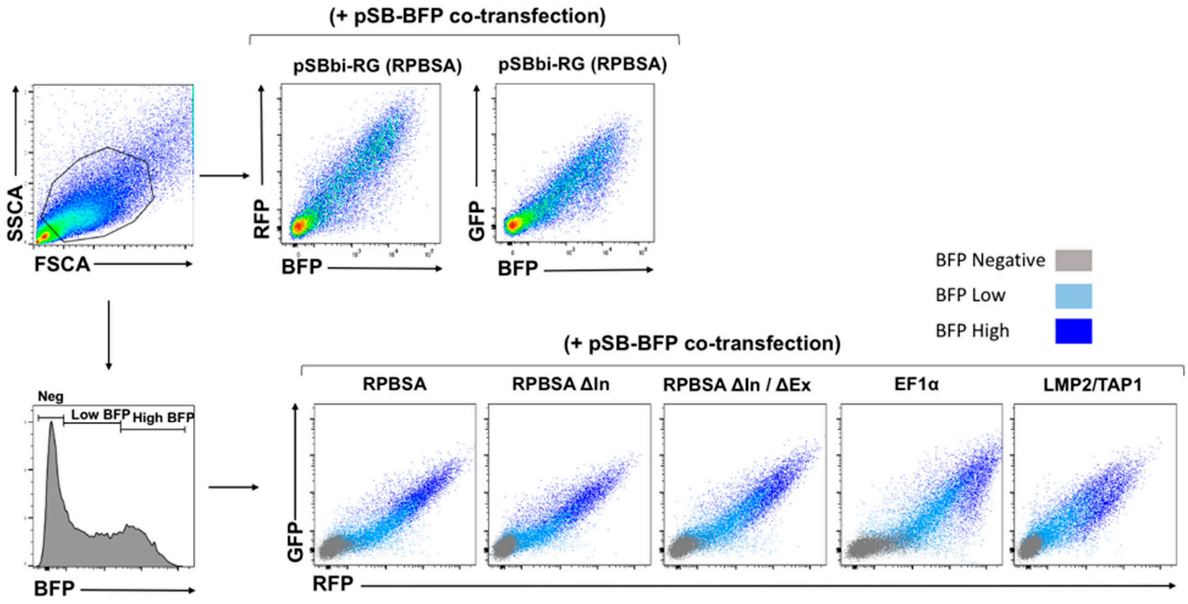

Figure S2. Co-transfection of pSB-BFP with bi-directional promoter constructs: To determine if the observed variable gene expression from bi-directional promoter activity was related to transfection efficiency or intrinsic promoter activity, we co-transfected HEK293 with bidirectional promoter constructs, together with a separate pSB-BFP plasmid in a 1:1 ratio. Negative (grey), low (light blue) and high (dark blue) BFP expressing cells are represented on the GFP vs. RFP plots for each promoter.

Figure S3

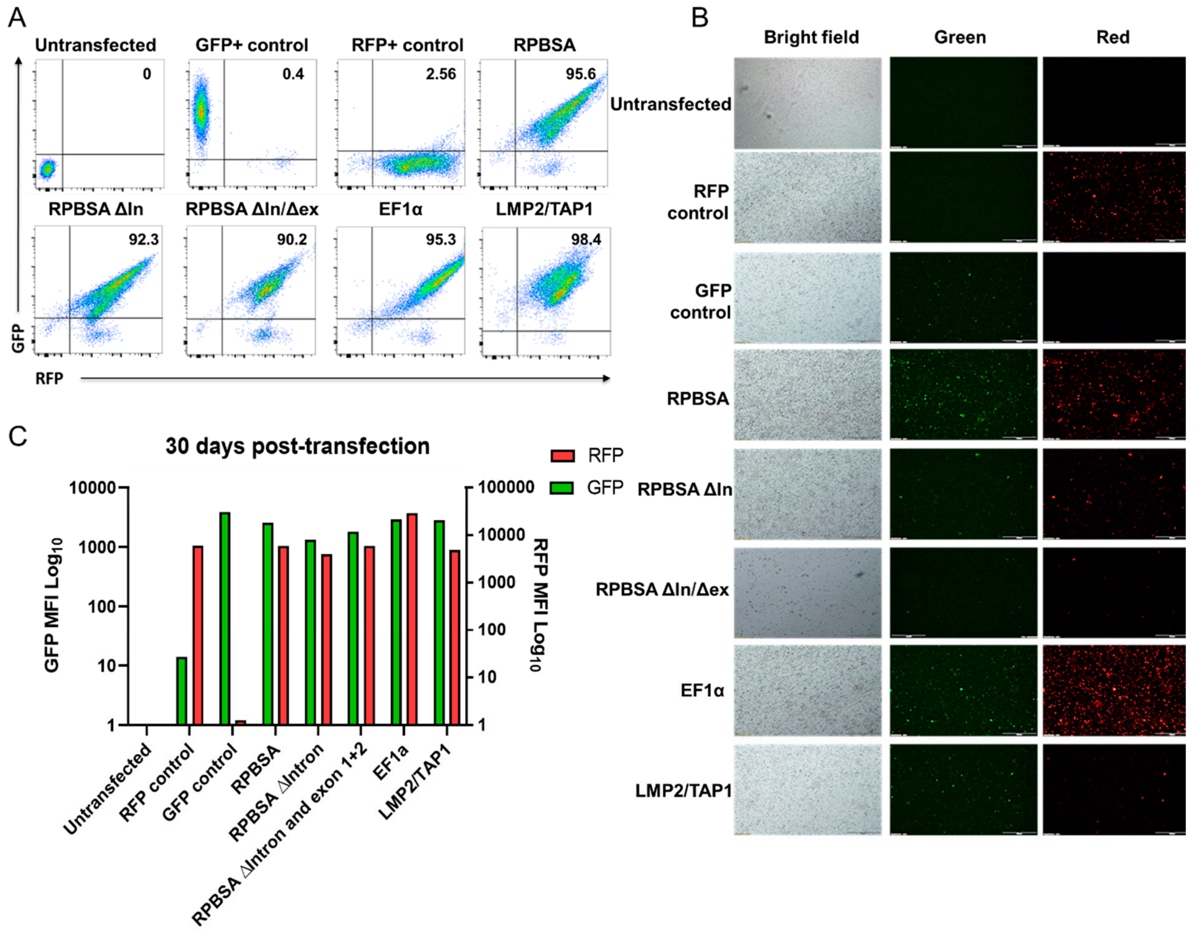

Figure S3. Monitoring maintenance of long-term expression by bidirectional promoters after 30 days. (A) Flow cytometry carried out to measure the expression GFP and RFP in Jurkat cells. Cells were subjected to FSc and SSc doublet discrimination (B) Fluorescent microscopy of Jurkat cells expressing GFP and RFP. (C) MFI assessment of Jurkat cells for GFP and RFP expression.
